# Prognostic factors in ICU patients with community-acquired bacterial meningitis: A post hoc analysis of a multicenter prospective cohort study

**DOI:** 10.1016/j.jointm.2026.04.007

**Published:** 2026-06-13

**Authors:** Valentin de Villiers de la Noue, Sarah Tubiana, Maryam Rontani, Marion Giry, Benjamine Sarton, Jean-David Pommier, Nicolas Terzi, Mathieu Jozwiak, Laurent Argaud, Claire Dupuis, Nathalie Marin, Jean-Pierre Quenot, Paul Nedelec, Magali Garcia, François Barbier, Khaldoun Kuteifan, Nacim Benchabane, Xavier Duval, Bruno Mourvillier, the COMBAT cohort study

**Affiliations:** 1 Unité de Médecine Intensive et Réanimation Polyvalente, CHU Reims, Reims, France; 2 Médecine Intensive Réanimation, Hôpital Européen Georges Pompidou, Paris, France; 3 Centre d’Investigation Clinique, Hôpital Bichat Claude Bernard, Paris, France; 4 Medical Intensive Care Unit, Normandie Université, UNIROUEN, UR 3830, CHU Rouen, Rouen, France; 5 Critical Care Unit, University Teaching Hospital of Purpan, Toulouse, France; 6 Réanimation Médicale et Chirurgicale, CHU de Guadeloupe, Chemin Chauvel, Les Abymes, Guadeloupe, France; 7 Medical Intensive Care Unit, University Hospital of Rennes, Rennes, France; 8 Service de Médecine Intensive Réanimation, CHU de Nice, Nice, France; 9 Medical Intensive Care Unit, Edouard Herriot Hospital, Hospices Civils de Lyon, Lyon, France; 10 Service de Médecine Intensive Réanimation, Université Clermont-Auvergne, Clermont-Ferrand, France; 11 Médecine Intensive Réanimation, APHP, Centre Université Paris Cité, Cochin Hospital, Paris, France; 12 Service de Médecine Intensive-Réanimation, Centre Hospitalier Universitaire Dijon, Dijon, France; 13 Nantes University, Nantes University Hospital, Medecine Intensive Reanimation, Nantes, France; 14 Virology Laboratory, CHU Poitiers, Poitiers, France; 15 Service de Médecine Intensive-Réanimation, Centre Hospitalier Régional d’Orléans, Orléans, France; 16 Service de Réanimation Médicale GHRMSA, Mulhouse, France; 17 Intensive Care Medicine Department, Lapeyronie Hospital, Montpellier University Hospital, Montpellier, France; 18 Unité Cardiovir, UMRS 1320, Université Reims Champagne Ardenne, Reims, France

**Keywords:** Meningitis, Bacterial, Community-acquired infections, Intensive care units, Prognosis, Risk factors

## Abstract

**Background:**

Community-acquired adult bacterial meningitis (CABM) is a severe condition associated with significant morbidity and mortality, particularly in patients requiring intensive care unit (ICU) admission. Previous studies have identified several risk factors for poor outcomes, including advanced age, immunosuppression, and delayed treatment. However, data on ICU-specific prognostic factors remain limited. This study aimed to identify factors associated with ICU admission, predictors of 30-day mortality, and long-term outcomes in ICU-admitted patients with CABM.

**Methods:**

This post hoc analysis utilized data from 27 ICUs from the COMBAT cohort, a prospective multicenter study in France from February 2013 to July 2015. The study included patients over 18 years old with confirmed CABM. Clinical, microbiological, and ICU-specific data, including Sequential Organ Failure Assessment (SOFA) scores, were collected. The primary outcome was 30-day mortality, and secondary outcomes included ICU admission predictors and 12-month functional status. Multivariable logistic regression models with pre-specified clinically relevant variables were used to identify factors associated with 30-day mortality and ICU admission; model performance was assessed using the c-statistic and Hosmer–Lemeshow goodness-of-fit test.

**Results:**

Of the 210 patients admitted to the ICU, 36 (17.1%) died within 30 days. Significant predictors of mortality included elevated SOFA scores at ICU admission and on day 3 in univariate and multivariate analyses. Age and non-meningococcal CABM were associated with mortality in the univariate analysis but not in the multivariate analysis. ICU admission was associated with altered consciousness and *Neisseria meningitidis* CABM according to univariate and multivariate analyses. In multivariable analysis, higher SOFA scores at admission (OR=1.49, 95% CI: 1.28 to 1.74) and on day 3 (OR=1.51, 95% CI: 1.28 to 1.78) were independently associated with 30-day mortality, while altered consciousness (OR=7.51, 95% CI: 4.65 to 15.42) and *N. meningitidis* identification in cerebrospinal fluid (OR=3.45, 95% CI: 1.15 to 10.00) were independently associated with ICU admission. At 12 months, 51.0% of survivors had poor outcomes on the Glasgow Outcome Scale, with significant neurofunctional impairments observed.

**Conclusion:**

This study identified key prognostic factors for ICU admission and 30-day mortality in CABM patients. Altered consciousness and *N. meningitidis* infection were associated with ICU admission, whereas elevated SOFA scores at admission and on day 3 were associated with mortality. Despite intensive care, mortality rates remain high, and long-term neurofunctional deficits are common among survivors.

**Trial registration:**

ClinicalTrials.gov identifier NCT01730690.

## Introduction

Community-acquired adult bacterial meningitis (CABM) is a rare disease with an annual incidence of approximately 1.57/100,000 inhabitants.^[^^1^^]^ The mortality rate associated with CABM varies from 9.2% to 30%, depending on the population and study.^[^[2], [3], [4], [5], [6]^]^

An unfavorable outcome, defined by a Glasgow Outcome Scale score ranging from 1 to 4 points, reached 34–43% of patients at discharge.^[^^2^^,^^6^^,^^7^^]^ It also leads to high morbidity, including neurological sequelae such as hearing or cognitive impairment and focal cerebral deficits.^[^^2^^,^^5^^,^[7], [8], [9]^]^ Studies have identified various risk factors, including age over 70 years, immunosuppression, low blood pressure, high heart rate, low Glasgow Coma Scale (GCS) score, cranial nerve palsy, high serum inflammatory markers, low white blood cell count in the cerebrospinal fluid (CSF), *Streptococcus pneumoniae* (*S. pneumoniae*) infection, delayed antimicrobial treatment, and lack of adjunctive dexamethasone.^[^^2^^,^^5^^,^^7^^,^[10], [11], [12], [13], [14], [15], [16], [17], [18], [19]^]^

Nevertheless, a limited number of studies have focused on intensive care unit (ICU) patients, although most cases of meningitis are admitted to the ICU because of their severity.^[^^4^^,^^6^^,^^20^^]^ To investigate this specific population, we conducted a post hoc analysis of a prospective multicenter French cohort of CABM patients that included both ICU-admitted and non-ICU patients, with ICU-admitted patients defined *a priori* as the subgroup of interest. We aimed to determine prognostic factors in this severe population. The main objective was to identify factors associated with 30-day mortality among patients admitted to the ICU. We also performed secondary analyses comparing ICU-admitted and non-ICU patients to identify factors associated with ICU admission, and described 12-month outcomes among ICU-admitted patients.

## Methods

### Study design and population

This study is a post hoc analysis based on data collected from the French multicenter cohort (COMBAT). COMBAT is a prospective study conducted from February 2013 to July 2015 across 69 hospitals that aimed to identify factors associated with unfavorable in-hospital outcomes (death or disability) in adults with CABM. In this post hoc study focusing on patients admitted to the ICU, cases from 27 ICUs were included because data from other centers were not readily available.

The inclusion criterion in this post hoc analysis was consecutive patients older than 18 years who presented with CABM or purpura fulminans admitted to the ICU. CABM was defined as having at least one of the following: (1) A positive CSF culture for bacteria; (2) A combination of CSF pleocytosis (defined by a CSF leukocyte count >5/mm^3^) with a positive blood culture or a positive CSF polymerase chain reaction (PCR) or antigen test for a meningitis-causing bacterium; (3) A combination of CSF pleocytosis with a culture or specific PCR from a skin biopsy in the case of petechiae.^[^^21^^]^

### Data collection

Clinical and microbiological data were prospectively collected, and strains were sent to the corresponding national reference centers.

Patients were followed up throughout hospitalization and were contacted by phone 12 months after enrolment. For participants lost to follow-up, vital status data were obtained from the French Epidemiology Centre on Medical Causes of Death database.

ICU-specific data (reasons for admission to the ICU, length of ICU stay, Sequential Organ Failure Assessment [SOFA] score, CSF findings, corticosteroid therapy, need for orotracheal intubation, temperature management, intracerebral device, electroencephalography, endocarditis, pneumonia, 30-day mortality, and cause of death) were retrospectively collected from hospital records.

### Outcome measures

For the ICU data, we used the SOFA score to assess severity.^[^^21^^]^ Altered mental status was defined as a GCS score strictly under 14, and coma status was defined as a GCS score ≤ 8.^[^^22^^]^ Ventilator-associated pneumonia (VAP) is defined as an infection of the pulmonary parenchyma in patients exposed to invasive mechanical ventilation (MV) for at least 48 h and is considered part of ICU-acquired pneumonia.^[^^23^^]^

Disability outcomes were assessed via the modified Rankin scale (mRS) and Glasgow Outcome Scale (GOS) 12 months after enrolment.^[^^24^^]^ Favorable outcomes were defined by a score of 0 (no symptoms at all) or 1 (no significant disability despite symptoms; able to carry out all usual duties and activities) on the mRS at discharge. A GOS of 5 (good recovery) was considered a favorable outcome.^[^^25^^]^ Depressive symptoms were assessed with the Center for Epidemiologic Studies Depression (CES-D) scale.^[^^26^^]^

Health-related quality of life (HRQL) was evaluated via the SF-12 Health Survey. An individual was defined as having impaired physical (or mental) HRQL if their physical component summary (or mental component summary) was lower than the 25th percentile of the score distribution of the score in the general French population of the same age group and gender, and an established approach was used to clinically interpret the results.^[^^27^^]^

### Statistical analysis

First, a descriptive analysis was performed on the population admitted to the ICU. Categorical variables are summarized as counts (percentages), and frequency distributions were compared via the chi-squared test or Fisher’s exact test, as appropriate. Continuous variables are expressed as medians with interquartile ranges (IQRs) and compared using Mann–Whitney *U* test.

Second, we searched for factors associated with 30-day mortality among patients admitted to the ICU via both univariate and multivariate analyses. To examine factors associated with 30-day mortality, only the following five clinically relevant variables were included: age, SOFA score at ICU admission, SOFA score on day 3, causative pathogen (*Neisseria meningitidis* [*N. meningitidis*] *vs.* others), and dexamethasone therapy. Associations were analyzed using a mixed-effects logistic regression model, with a subject-level random intercept to account for the correlation between repeated SOFA measurements. Multicollinearity was assessed using variance inflation factors (VIF). Sensitivity analysis used ΔSOFA (day 3 minus admission) in a logistic regression model to confirm the consistency of the results. Model performance was evaluated with the c-statistic (95% CI) for discrimination and the Hosmer–Lemeshow goodness-of-fit test for calibration. Third, we also aimed to identify factors associated with admission to the ICU via the same approach. Finally, a descriptive analysis of the 12-month patient characteristics was performed.

Missing data were not replaced. All the statistical tests were two-tailed, and *P* values <0.05 were considered statistically significant. Analyses were performed via SAS version 9.4 software (SAS Institute Inc., Cary, NC, USA).

## Results

### Patient characteristics

A total of 280 patients with bacterial meningitis were admitted to these 27 centers, 210 of whom were admitted to the ICU (Figure 1). Patients had a median age of 58.2 years (IQR: 37.9–68.0), and females accounted for 43.8% of the cohort. Previously published risk factors for meningitis were identified in 135 out of 210 (64.3%) patients (Table 1). Microbiological culture identified *N. meningitidis* in 45 (21.4%) patients and other microorganisms (predominantly *S. pneumoniae*) in 159 (75.7%); CSF culture was unavailable in 6 (2.9%) patients.

**Figure 1:**
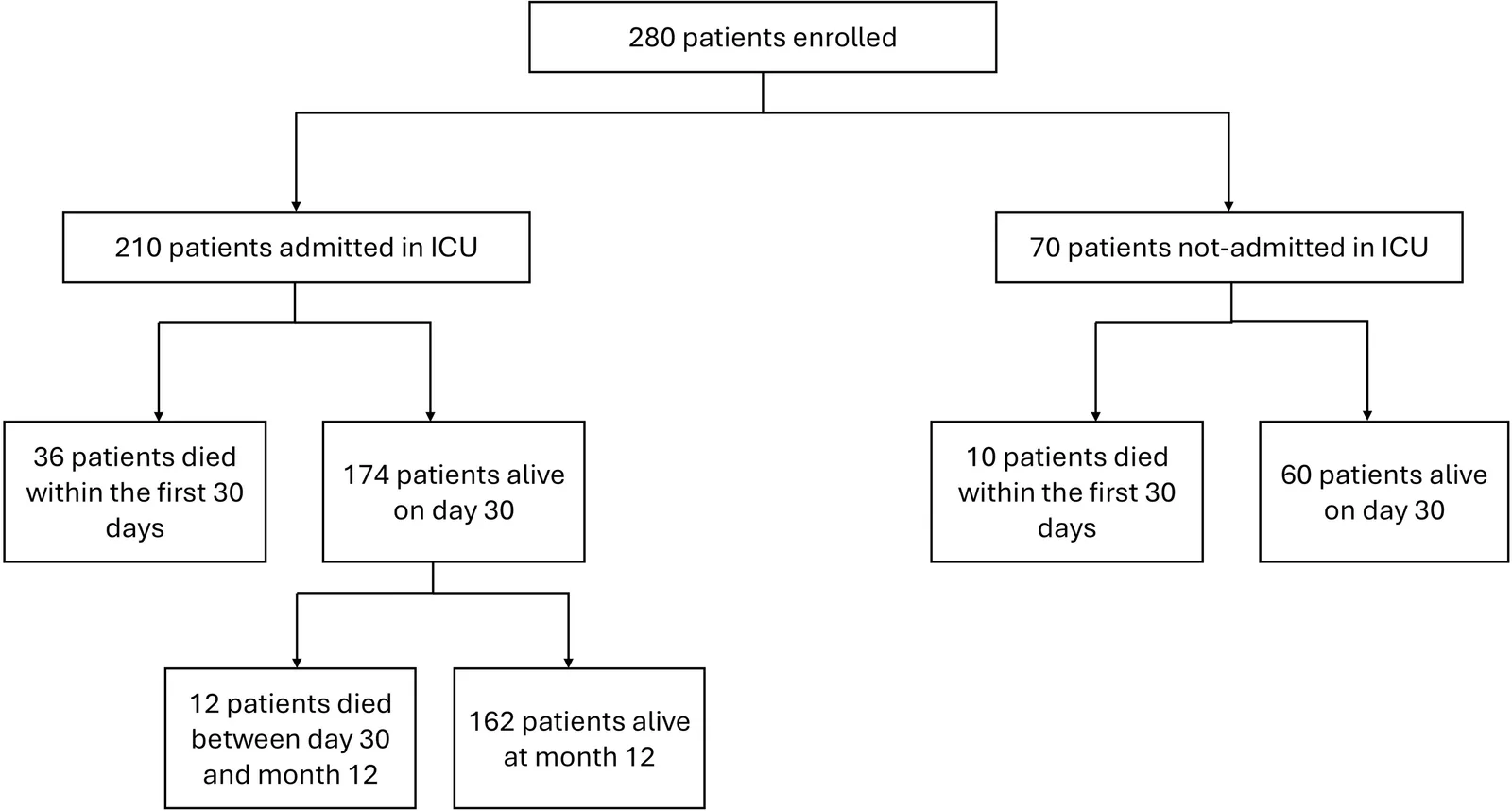
Flow chart of the study. ICU, Intensive care unit.

**Table 1:** Characteristics and outcomes of patients admitted to ICU (*n*=210).

Characteristics	Data
Background characteristics	
Age (years)	58.2 (37.9–68.0)
Female sex	92 (43.8)
Risk factor(s) for meningitis	135 (64.3)
Reason(s) for admission in ICU (*n*=210)	
Altered mental status	153 (72.9)
Hemodynamic failure	22 (10.5)
Respiratory distress	16 (7.6)
Sepsis or septic shock	4 (1.9)
Purpura	12 (5.7)
Acute renal failure	2 (1.0)
Seizure	4 (1.9)
Cardiorespiratory arrest	1 (0.5)
Patient monitoring (no failure)	16 (7.6)
SOFA score on admission	4.0 (2.0–8.0)
Length of ICU stay (days)	5.0 (2.0–8.0)
Cerebrospinal fluid findings at admission	
White cell counts (cells per mm^3^)	2000.0 (364.0–5200.0)
Protein (g/L)	4.8 (2.6–7.4)
Glucose (mmol/L)	0.2 (0.0–2.6)
SOFA score on day 3	5.0 (2.0–7.0)
Corticosteroid therapy (*n*=190)	151 (79.5)
Dexamethasone	142 (74.7)
HC/FC	9 (4.7)
Need for orotracheal intubation (*n*=206)	114 (55)
Duration of MV	5.0 (3.0–11.0)
Norepinephrine requirement (*n*=210)	73 (34.8)
Hypertonic saline osmotherapy (*n*=209)	4 (1.9)
Mannitol osmotherapy (*n*=209)	10 (4.8)
Targeted temperature management (*n*=207)	10 (4.8)
Intracranial pressure monitoring (*n*=207)	15 (7.2)
External ventricular drainage (*n*=207)	9 (4.3)
Electroencephalography (*n*=209)	131 (66.5)
Clinical course	
Endocarditis (*n*=209)	2 (1.0)
Pneumonia (excluding VAP) (*n*=198)	12 (6.1)
VAP (*n*=198)	20 (10.1)
Death within 30 days (*n*=209)	36 (17.1)
Causes of death	
Multiple organ dysfunction (*n*=35)	13 (37.1)
Decision to withdraw life support (*n*=35)	12 (34.3)
Brain death (*n*=36)	11 (30.6)

Data are presented as the *n* (%) or median (IQR).

FC, Fludrocortisone; HC, Hydrocortisone; ICU, Intensive care unit; IQR, Interquartile range; SOFA, Sequential Organ Failure Assessment; VAP, Ventilator-associated pneumonia.

### Factors associated with 30-day mortality in ICU patients

In our cohort, the 30-day mortality rate was 17.1% (Table 1). According to the univariate analysis, mortality was significantly associated with older age (67.7 years *vs.* 56.2 years, *P* <0.001) and respiratory distress at ICU admission (19.4% *vs.* 5.2%, *P*=0.009). The regression coefficients associated with age did not indicate a log-linear trend. Patients admitted for hemodynamic failure (19.4% *vs*. 8.6%, *P*=0.070) and sepsis (13.9% *vs*. 5.2%, *P*=0.070) tended toward increased mortality. *N. meningitidis* was associated with a greater survival rate on day 30 than were the other bacteria (5.6% *vs*. 25.6%, *P*=0.018), and other microorganisms were correspondingly more frequent among non-survivors (94.4% *vs*. 74.4%; *P*=0.018, from the same 2 × 2 chi-squared test), indicating greater 30-day survival in meningococcal CABM. Non-survivors had significantly higher SOFA scores at both admission (median 8.0 *vs*. 4.0, *P* <0.001) and day 3 (median 9.5 *vs*. 4.0, *P* <0.001). Supportive care measures were associated with 30-day mortality in the univariate analysis. Norepinephrine use was significantly more common in nonsurvivors (77.8% *vs*. 25.9%, *P* <0.001). Orotracheal intubation was almost universal in nonsurvivors (97.1% *vs*. 46.8%, *P* <0.001), although the use of coma as an indicator for intubation was not significantly different between the groups (70.6% *vs*. 62.0%, *P*=0.380). The use of hypertonic saline (8.3% *vs*. 0.6%, *P*=0.020), mannitol osmotherapy (13.9% *vs*. 2.9%, *P*=0.020), and external ventricular drainage was also more common in the non-survivors (14.3% *vs*. 2.3%, *P*=0.008). Dexamethasone was administered to 63.3% of non-survivors and 76.9% of survivors, with no statistically significant association with 30-day mortality (*P*=0.120). Hydrocortisone and fludrocortisone (HC/FC) use was more common in non-survivors (13.3% *vs*. 3.1%, *P*=0.030) (Table 2).

**Table 2:** Univariate analysis of factors associated with 30-day mortality among patients admitted to ICU (*n*=210).

Variables	Non-survivor (*n*=36)	Survivor (*n*=174)	*P* value
Background characteristics			
Age	67.7 (55.2–78.5)	56.2 (34.7–66.4)	0.001
Female sex	16 (44.4)	76 (43.7)	0.930
Risk factor(s) for meningitis	23 (63.9)	112 (64.3)	0.960
Reason(s) for admission in ICU			
Hemodynamic failure	7 (19.4)	15 (8.6)	0.070
Respiratory distress	7 (19.4)	9 (5.2)	0.009
Sepsis	5 (13.9)	9 (5.2)	0.070
Septic shock	1 (2.8)	2 (1.1)	0.430
Purpura	1 (2.8)	11 (5.2)	0.700
Acute renal failure	0 (0.0)	2 (1.1)	1.000
Altered mental status	11 (30.6)	128 (73.6)	0.610
Seizure	0 (0)	4 (2.3)	1.000
Cardiorespiratory arrest	1 (2.8)	0 (0)	0.170
Patient monitoring (no failure)	1 (2.8)	15 (8.6)	0.320
Microbiological culture on CSF			0.018
*Neisseria meningitidis*	2/36 (5.6)	43/168 (25.6)	
Other microorganisms	34/36 (94.4)	125/168 (74.4)	
Length of stay in ICU (days)	7.5 (3.5–16.5)	4.0 (2.0–7.0)	0.300
SOFA score on admission	8.0 (6.0–12.5)	4.0 (2.0–6.0)	<0.001
SOFA score on day 3	9.5 (6.0–14.0)	4.0 (1.0–6.0)	<0.001
Corticosteroid therapy	23/30 (76.7)	128/160 (80.0)	0.680
Dexamethasone	19/30 (63.3)	123/160 (76.9)	0.120
HC/FC	4/30 (13.3)	5/160 (3.1)	0.030
Electroencephalography	26/36 (72.2)	105/161 (65.2)	0.420
Pneumonia (excluding VAP)	3 (8.3)	9 (5.6)	0.460
Norepinephrine requirement	28 (77.8)	45 (25.9)	<0.001
Hypertonic saline osmotherapy	3 (8.3)	1 (0.6)	0.020
Mannitol osmotherapy	5/36 (13.9)	5/173 (2.9)	0.020
Targeted temperature management	3/35 (8.6)	7/172 (4.1)	0.380
Need for orotracheal intubation	34/35 (97.1)	80/171 (46.8)	<0.001
Reason(s) for intubation			
Altered mental status without coma	4/34 (11.8)	15/79 (19.0)	0.350
Coma	24/34 (70.6)	49/79 (62.0)	0.380
Respiratory distress	7/34 (20.6)	9/79 (11.4)	0.240
Hemodynamic failure	1/34 (2.9)	4/79 (5.1)	1.000
Seizure	0/34 (0)	3/79 (3.8)	0.550
Cardiorespiratory arrest	2/34 (5.9)	1/79 (1.3)	0.210
Duration of MV	8.0 (4.0–16.0)	4.0 (2.5–9.0)	0.780
VAP	6 (16.6)	14/162 (8.7)	0.230
Intracranial pressure monitoring	4/35 (11.4)	11/172 (6.4)	0.290
External ventricular drainage	5/35 (14.3)	4/172 (2.3)	0.008

Data are presented as the *n* (%) or median (IQR).

CSF, Cerebrospinal fluid; FC, Fludrocortisone; HC, Hydrocortisone; ICU, Intensive care unit; IQR, Interquartile range; SOFA, Sequential Organ Failure Assessment; VAP, Ventilator-associated pneumonia.

Multivariate analysis did not show a significant association between a lower 30-day mortality and age (*P*=0.160) or CABM caused by *N. meningitidis* (OR=0.08, 95% CI: 0.006 to 1.20, *P*=0.070). In contrast, higher SOFA scores at admission (OR=1.49, 95% CI: 1.28 to 1.74, *P* <0.001) and on day 3 (OR=1.51, 95% CI: 1.28 to 1.78, *P* <0.001) were independently associated with 30-day mortality. Dexamethasone therapy was not significantly associated with a decrease in mortality in the multivariable model (Table 3). The main multivariable model (Table 3) showed good discrimination (c-statistic 0.90, 95% CI: 0.87 to 0.94) and calibration (Hosmer–Lemeshow *P* >0.05), with low multicollinearity (all VIF <3). Sensitivity analysis using ΔSOFA confirmed the robustness of these findings N (Supplementary Table S1).

**Table 3:** Multivariate analysis of factors associated with 30-day mortality among patients admitted to ICU (*n*=210).

Variables	OR (95% CI)	*P* value
SOFA score on admission	1.49 (1.28–1.74)	0.001
SOFA score on day 3	1.51 (1.28–1.78)	<0.001
Dexamethasone corticosteroid therapy	0.52 (0.15–1.80)	0.300
Microbiological culture on CSF		0.070
*Neisseria meningitidis vs.* other bacteria	0.08 (0.006–1.20)	
Age group		0.160
<35 years	Ref.	
35–65 years	0.96 (0.12–7.18)	
≥65 years	2.65 (0.34–20.56)	

Model performance: Area Under Curve=0.90 (95% CI: 0.87–0.94); Hosmer–Lemeshow test, *P* >0.05.

Collinearity: The VIF values were 1.25 for admission SOFA, 1.26 for day 3 SOFA, 1.02 for dexamethasone therapy, 1.59 for causative pathogen, and ranged from 2.68 to 2.94 for age groups.

CSF, Cerebrospinal fluid; ICU, Intensive care unit; SOFA, Sequential Organ Failure Assessment; VIF, Variance inflation factor.

### Factors associated with ICU admission

According to the univariate analysis, the ICU group was significantly younger than the non-ICU group (median age 58.2 years *vs*. 61.5 years, *P*=0.040), whereas the proportion of female patients did not differ significantly (43.8% *vs*. 47.1%, *P*=0.630). Risk factors for bacterial meningitis did not differ significantly between the ICU and non-ICU groups (64.3% *vs*. 69.6%, *P*=0.420).

Significantly more patients in the ICU group presented with altered consciousness (80.9% *vs*. 45.6%, *P* <0.001) and purpura (11.4% *vs*. 0%, *P*=0.003). There was no significant difference between the groups in terms of prior influenza-like illness, previous antibiotic use, or seizures before admission. Patients with negative direct smear results were significantly less likely to be admitted to the ICU.

In univariate analysis, the proportion of *N. meningitidis* did not differ significantly between ICU and non-ICU groups (21.4% *vs*. 15.7%; *P*=0.270), nor did the proportion of other microorganisms (75.7% *vs.* 82.9%; *P*=0.270, from the same 2 × 2 chi-squared test) (Table 4).

**Table 4:** Univariate analysis of factors associated with admission to ICU (*n*=280).

Variables	Admission to ICU (*n*=210)	No admission to ICU (*n*=70)	*P* value
Age	58.2 (37.9–68.0)	61.5 (53.5–68.6)	0.040
Female sex	92 (43.8)	33 (47.1)	0.630
Bacterial meningitidis risk factors	135/210 (64.3)	48/69 (69.6)	0.420
Microbiological culture on CSF (n=273)			0.270
*Neisseria meningitidis*	45/210 (21.4)	11/70 (15.7)	
Other microorganisms	159/210 (75.7)	58/70 (82.9)	
Initial presentation			
Influenza-like illness in the 15 days prior to meningitis	81/205 (39.5)	26/67 (38.8)	0.920
Prior treatment with antibiotics	81/207 (39.1)	22/68 (32.4)	0.320
Seizure before admission	21/207 (10.1)	4/69 (5.8)	0.280
Alteration of consciousness	169/209 (80.9)	31/68 (45.6)	<0.001
Presence of other infectious outbreaks	86 (41.0)	27 (38.6)	0.730
Focal neurological symptoms	68 (32.4)	25 (35.7)	0.610
Purpura	24 (11.4)	0	0.003

Data are presented as the *n* (%) or median (IQR).

CSF, Cerebrospinal fluid; ICU, Intensive care unit; IQR, Interquartile range.

Multivariate analysis indicated that identification of *N. meningitidis* in CSF culture and alteration of consciousness were significantly associated with ICU admission (OR=3.45, 95% CI: 1.15 to 10.00, *P*=0.028 and OR=7.51, 95% CI: 4.65 to 15.42, *P* <0.001, respectively). This multivariable model for ICU admission showed acceptable discrimination (c-statistic 0.70, 95% CI: 0.63 to 0.77) and adequate calibration (Hosmer–Lemeshow goodness-of-fit *P*=0.480).

### Twelve-month follow-up outcomes

At the 12-month follow-up of the 210 ICU-admitted patients, 49.0% (74/151) achieved a favorable GOS score of 5, and 60.1% (92/153) had favorable outcomes according to the mRS score (0 or 1) at 12 months. (Table 5). Among the 162 patients who survived to 30 days, these proportions increased to 71.8% for the GOS and 62.9% for the mRS. Hearing loss (Hearing Handicap Inventory (HHI) ≥ 9) was reported in 30.9% of patients. Return to work was achieved by 47.1% of patients overall, with significant age-related differences: 79.2% in those under 55 years *vs.* 18.5% in older patients in univariate analysis (*P* <0.001). The use of tranquilizers or sleeping pills was reported in 14.6% of patients before admission and in 11.3% after meningitis, whereas antidepressant use was reported in 7.2% before admission and 10.4% after meningitis. Depressive symptoms, assessed using the CES-D, were present in 34.0% of patients. Impaired physical and mental component scores on the SF-12 were observed in 52.0% and 31.0% of patients, respectively.

**Table 5:** Patients’ outcomes at the 12-month follow-up.

Outcomes	Data
Among patients admitted to ICU (*n*=210)	
Favorable GOS (=5)	74/151 (49.0)
Favorable modified Rankin disability score (=0,1)	92/153 (60.1)
Among patients admitted to ICU who survived at least 30 days (*n*=162)	
Resumption of professional activity	48/102 (47.1)
Use of tranquilizers or sleeping pills prior to meningitis	22/151 (14.6)
Use of tranquilizers or sleeping pills	12/106 (11.3)
Antidepressant medication prior to meningitis	11/152 (7.2)
Antidepressant medication	11/106 (10.4)
Hearing loss (HHI score ≥ 9)	32/104 (30.9)
Favorable GOS (=5)	74/103 (71.8)
Favorable modified Rankin disability score (=0,1)	66/105 (62.9)
Depressive symptoms (CES-D score ≥ 23 for women and ≥ 17 for men)	34/100 (34.0)
Alteration of SF-12 physical health score	52/100 (52.0)
Alteration of SF-12 mental health score	31/100 (31.0)

Data are presented as the *n* (%) or median (IQR).

CES-D, Center for Epidemiologic Studies Depression; GOS, Glasgow Outcome Scale; ICU, Intensive care unit; IQR, Interquartile range.

## Discussion

To our knowledge, our study is among the few specifically addressing the ICU course of patients suffering from CABM admitted to critical care, and provides one of the most comprehensive prospective assessments of 12-month outcomes in this population – combining functional, psychological, quality-of-life, and occupational measures.

We observed substantial long-term morbidity, with nearly half of the patients presenting with poor functional outcomes and high rates of hearing loss, depressive symptoms, and impaired quality of life. Return to work was achieved by less than half of the patients, with a marked difference between age groups. More specifically, depressive symptoms (CES-D) were identified in 34.0% of survivors, physical HRQL (SF-12) was impaired in 52.0%, and professional reintegration was achieved by only 47.1% of patients – with a striking age-dependent gradient: 79.2% in patients aged under 55 years *vs.* only 18.5% in older patients (*P* <0.001). Data on long-term sequelae in CABM survivors are very limited, and none have specifically focused on patients requiring ICU management, who represent the most severe forms of the disease. The prevalence of cognitive impairment appeared to be almost twice as high in our cohort than in a slightly smaller cohort of adults with pneumococcal meningitis.^[^^28^^]^ This difference does not appear to be explained only by the ICU environment, as in our experience and the literature, cognitive impairment was also lower in miscellaneous ICU patients.^[^^29^^,^^30^^]^ In addition, our study combines multiple validated outcomes – including the GOS, mRS, CES-D, SF-12, and professional reintegration – and offers a comprehensive overview of the physical, psychological, and social consequences 1 year after ICU discharge.

The lower rate of favorable outcomes on the mRS compared to the GOS (62.9% *vs.* 71.8%) likely reflects the greater stringency of the mRS definition, which requires complete absence of symptoms or disability, whereas the GOS captures the ability to resume normal life more broadly. Hearing loss was observed in 30.9% of survivors at 12 months, a rate consistent with the 33% reported at hospital discharge in a large prospective ICU cohort,^[^^6^^]^ suggesting that auditory sequelae largely persist without meaningful recovery beyond the acute phase. Regarding quality of life, physical impairment was more prevalent than mental impairment (52.0% *vs.* 31.0% on the SF-12), suggesting that somatic sequelae predominate over psychological ones in the 1st year. Finally, the modest increase in antidepressant use (7.2% before *vs.* 10.4% after meningitis) contrasts with the 34.0% prevalence of depressive symptoms on the CES-D, suggesting a substantial gap between the burden of depression and its pharmacological recognition and treatment in this population. A comparison of long-term outcomes between ICU-admitted patients with CABM and those with other severe infections, such as sepsis or pneumonia, would be valuable for assessing which sequelae are specific to meningitis. These findings highlight the lasting impact of CABM in ICU survivors beyond the acute phase and support the need for structured follow-up strategies in line with current recommendations for the management of postintensive care syndrome (PICS), including neurological, psychological, audiological, and functional assessment, as well as social and occupational reintegration.^[^^31^^]^

With respect to pathogens, we identified a lower mortality rate in patients with CABM caused by *N. meningitidis* via univariate analysis than in those with other bacteria, whereas *N. meningitidis* was linked to ICU admission in both univariate and multivariate analyses. This finding emphasizes the pathogen-specific variability in CABM outcomes and highlights the relative virulence of *S. pneumoniae* and other nonmeningococcal bacteria. A higher *S. pneumoniae* burden has previously been described in the literature, and our findings additionally suggest an association between *N. meningitidis* and ICU admission.^[^^6^^]^ The presence of purpura, which is more commonly associated with *N. meningitidis* CABM, may have prompted clinicians to initiate antimicrobial therapy prior to a definitive diagnosis and admit patients to a critical care unit.^[^^32^^]^ Delays in antibiotic administration are linked to unfavorable outcomes at discharge.^[^^33^^]^ Unfortunately, we were unable to assess the time interval between admission, diagnosis, and antibiotic therapy initiation to estimate the role of quick antibiotic administration in the lower mortality rate.

Our cohort included both patients with confirmed bacterial meningitis and those with purpura related to meningococcal sepsis. This reflects real-life ICU practice, where admission is driven by clinical severity rather than microbiological confirmation alone.

Beyond these long-term findings, the present study also provides insights into short-term prognostic factors, though these should be interpreted in the context of the limited number of events.

The study also highlighted the prognostic value of the SOFA score. It has already been established that the SOFA score at ICU admission is associated with mortality.^[^^4^^]^ However, few studies have specifically evaluated the association between SOFA score and mortality in CABM patients on day 3 (OR=1.51 [1.28–1.78]). These findings suggest that early and persistent organ dysfunction is a critical determinant of outcome. This finding emphasizes the need for intensivists to closely monitor organ function as part of an early warning system, which may result in more aggressive interventions in patients with higher scores. The SOFA score is a general indicator of organ failure severity and has been extensively validated in various ICU populations, including meningitis.^[^^34^^]^ Our results support its relevance in this setting, and to our knowledge, these data are among the first to suggest that SOFA score dynamics, particularly on day 3, are independently associated with 30-day mortality in CABM patients. This underlines the importance of repeated organ function assessments beyond the initial ICU admission. Our findings partially differ from those of previous ICU studies on CABM, which have often identified predictors such as coma at admission, purpura, or septic shock as strong indicators of poor outcomes.^[^^2^^,^^4^^,^^6^^]^ In our cohort, these variables were not significantly associated with 30-day mortality, even in the univariate analysis, and were therefore not included in the multivariate model. This discrepancy may reflect differences in patient selection, case definitions, or healthcare organization between studies. A lack of statistical power may also have contributed to these negative findings. These results underscore the importance of re-evaluating classical prognostic markers in contemporary ICU cohorts via standardized definitions and sufficiently powered studies.

The correlation between respiratory distress and hemodynamic failure with increased mortality further reflects the intricate nature of CABM management in ICU patients. Despite the implementation of intensive supportive care measures such as norepinephrine administration, these disorders are associated with a high mortality rate. In nonsurvivors, the more frequent use of HC/FC reflects their administration in response to septic shock.^[^^35^^,^^36^^]^ Nevertheless, our data suggest that even with these interventions, mortality remains high, underscoring the necessity for innovative therapeutic approaches or early detection of patients who may not benefit from conventional treatments.

This study did not identify a significant reduction in 30-day mortality with the administration of corticosteroids, including dexamethasone. However, the study was not designed to assess corticosteroid efficacy, and these findings are subject to significant confounding by indication; the trend is consistent with previous studies suggesting a benefit of early corticosteroid use.^[^[17], [18], [19]^]^ Patients who received HC/FC had higher 30-day mortality than did those treated with dexamethasone. However, all patients in the HC/FC group were in shock, a known condition that worsens the prognosis. Unfortunately, no conclusion can be drawn regarding the comparative efficacy of dexamethasone *vs.* HC/FC in CABM patients with septic shock.

A key strength of our study is the large, multicenter cohort, which enhances the generalizability of our findings across diverse healthcare settings. The prospective data collection and use of standardized SOFA scoring add rigor to our analysis, allowing for robust and comparable outcomes. Additionally, long-term follow-up provides valuable insights into the chronic impact of CABM, a dimension often underexplored in acute care studies.

However, several limitations must be acknowledged. The post hoc nature of this analysis introduces potential selection bias as the original study was not designed to address the specific hypotheses tested here. The secondary analysis of factors associated with ICU admission should be interpreted with caution, as ICU admission may have been influenced by center-level practices or bed availability rather than strict application of French guidelines, and was limited to 27 out of 69 centers, limiting external validity. Similarly, interpretation of corticosteroid therapy requires caution, as HC/FC was mainly administered to patients with septic shock, reflecting confounding by indication, and the timing of dexamethasone was not recorded. Due to limited statistical power, we did not detect an association between age and mortality, unlike other studies.^[^^7^^]^ However, the multivariate OR for dexamethasone therapy and 30-day mortality among ICU patients was consistent with prior reports.^[^^2^^,^^18^^]^

Regarding mortality interpretation, among the 36 deaths recorded, 12 (34.3%) resulted from decisions to withdraw life-sustaining therapy (WLST), which limits the interpretability of mortality associations, as WLST decisions are influenced by factors not captured in the multivariable model.

This analysis was performed 8 years after the end of the cohort inclusion. Although CABM-specific guidelines and therapeutics have not substantially changed since enrolment, the management of sepsis has evolved considerably over the past decade, introducing a risk of time-lag bias, particularly for mortality outcomes related to organ failure. Despite this limitation, all outcomes were collected prospectively, and the scarcity of meningitis-specific ICU data made it reasonable to use these data.

## Conclusions

Our study identified critical prognostic factors for ICU admission and death within 30 days in patients with CABM. Altered consciousness and the presence of meningococcal CABM were independently associated with ICU admission, and an elevated SOFA score on day 3 was independently associated with mortality within 30 days. Nonmeningococcal CABM and age were associated with increased mortality in the univariate analysis but not in the multivariate analysis. Even with the aggressive use of supportive care, the mortality rate remains high, and long-term survivors often experience significant neurofunctional impairment. These findings underscore the importance of early recognition, appropriate ICU admission, and a customized approach to managing severe cases of CABM to improve both short-term and long-term outcomes. Further research is needed to optimize therapeutic and rehabilitation strategies.

## COMBAT Study Group

Principal investigator: Xavier DUVAL.

Steering committee: Bruno HOEN, Bruno MOURVILLIER, Marie-Cécile PLOY, Sarah TUBIANA, Emmanuelle VARON.

Scientific committee: steering committee and the following members: François CARON, Pierre-Edouard BOLLAERT, Olivier GAILLOT, Muhamed-Kheir TAHA, Claire POYART, Stephane BONACORSI, François VANDENESCH, Emmanuelle CAMBAU, Marc LECUIT, Alain GRAVET, Bruno FRACHET, Thomas De BROUCKER, Daniel LEVY BRUHL, François RAFFI, Marie PREAU.

COMBAT clinical centers: Nadia ANGUEL, Laurent ARGAUD, Sophie ARISTA, Laurence ARMAND-LEFEVRE, Stéphanie BALAVOINE, Régine BARADUC, Guilène BARNAUD, Guillaume BERAUD, Louis BERNARD, Georges BERNARS, Dominique BERTEI, Emilie BESSEDE, Typhaine BILLARD POMARES, Charlotte BIRON, Stéphane BLAND, Julien BOILEAU, Patrice BOUBEAU, Sandra BOURDON, Aurore BOUSQUET, Sophie BOYER, Alexis BOZORG-GRAYELI, Laurent BRET, Cédric BRETONNIERE, François BRICAIRE, Elsa BROCAS, Michel BRUN, Jennifer BURET, Christophe BURUCOA, Jean CABALION, Mathieu CABON, Emmanuelle CAMBAU, Guillaume CAMUSET, Christophe CANEVET, François CARON, Anne CARRICAJO, Bernard CASTAN, Eric CAUMES, Charles CAZANAVE, Amélie CHABROL, Thibaut CHALLAN-BELVAL, Vanessa CHANTEPERDRIX-MARILLIER, Chantal CHAPLAIN, Caroline CHARLIER-WOERTHER, Hélène CHAUSSADE Catherine CHIROUZE, Bernard CLAIR, Julien COLOT, Jean-Marie CONIL, Hugues CORDEL, Philippe CORMIER, Joël COUSSON, Pierrick CRONIER, Eric CUA, Anne DAO-DUBREMETZ, Sylvie DARGERE, Nicolas DEGAND, Sophie DEKEYSER, Deborah DELAUNE, Eric DENES, Pierre-Francois DEQUIN, Diane DESCAMPS, Elodie DESCLOUX, Jean-Luc DESMARETZ, Jean-Luc DIEHL, Jérôme DIMET, Aurélien DINH, Xavier DUVAL, Lelia ESCAUT, Claude FABE, Frédéric FAIBIS, Clara FLATEAU, Nathalie FONSALE, Emmanuel FORESTIER, Nicolas FORTINEAU, Amandine GAGNEUX-BRUNON, Caroline GARANDEAU, Magali GARCIA, Denis GAROT, Stéphane GAUDRY, François GOEHRINGER, Alain GRAVET, Valérie GREGOIRE-FAUCHER, Marine GROSSET, Camélia GUBAVU, Isabelle GUEIT, Dominique GUELON, Thomas GUIMARD, Jérôme GUINARD, Tahar HADOU, Jean-Pierre HELENE, Sandrine HENARD, Benoit HENRY, Anne-Cécile HOCHART, Bruno HOEN, Gabriela ILLES, Sylvain JAFFUEL, Irène JARRIN, Françoise JAUREGUY, Cédric JOSEPH, Marie-Emmanuelle JUVIN, Samer KAYAL, Solen KERNEIS, LACASSIN, Isabelle LAMAURY, Philippe LANOTTE, Etienne LAURENS, Henri LAURICHESSE, Cécile LE BRUN, Vincent LE MOING, Paul LE TURNIER, Hervé LECUYER, Sylvie LEDRU, Céline LEGRIX, Adrien LEMAIGNEN, Chantal LEMBLE, Ludovic LEMEE, Olivier LESENS, Marion LEVAST, Claire LHOMMET, Silvija MALES, Edith MALPOTE, Guillaume MARTIN-BLONDEL, Matthieu MARX, Raphael MASSON, Olivier MATRAY, Aurore MBADI, Frédéric MECHAI, Guillaume MELLON, Audrey MERENS, Marie Caroline MEYOHAS, Adrien MICHON, Joy MOOTIEN YOGANADEN, David MORQUIN, Stéphane MOULY, Natacha MROZEK, Sophie NGUYEN, Yohan NGUYEN, Maja OGIELSKA, Eric OZIOL, Bernard PAGE, Solène PATRAT-DELON, Isabelle PATRY, André PECHINOT, Sandrine PICOT, Denys PIERREJEAN, Lionel PIROTH, Claire PLASSART, Patrice PLESSIS, Marie-Cécile PLOY, Laurent PORTEL, Patrice POUBEAU, Marie POUPARD, Claire POYART, Thierry PRAZUCK, Luc QUAESAET, François RAFFI, Adriatsiferana RAMANANTSOA, Christophe RAPP, Laurent RASKINE, Josette RAYMOND, Matthieu REVEST, Agnès RICHE, Stéphanie ROBADAY-VOISIN, Frédéric ROBIN, Jean-Pierre ROMASZKO, Florence ROUSSEAU, Anne-Laure ROUX, Cécile ROYER, Matthieu SAADA, Dominique SALMON, Carlo SAROUFIM, Jean Luc SCHMIT, Manuela SEBIRE, Christine SEGONDS, Valérie SIVADON-TARDY, Nathalie SOISMIER, Olivia SON, Simon SUNDER, Florence SUY, Didier TANDE, Jacques TANKOVIC, Nadia VALIN, Nicolas VAN GRUNDERBEECK, François VANDENESCH, Emmanuelle VARON, Renaud VERDON, Michel VERGNAUD, Véronique VERNET-GARNIER, Magali VIDAL, Virginie VITRAT, Daniel VITTECOQ, Fanny VUOTTO.

Coordination and statistical analyses (Clinical trial unit, Hôpitaux Universitaires Paris Nord Val de Seine, AP-HP, Paris): Isabelle GORENNE, Cédric LAOUENAN, Estelle MARCAULT, France MENTRE, Blandine PASQUET, Carine ROY, Sarah TUBIANA.

## Acknowledgments

The authors would like to thank Sarrah Sellam, Jean-Luc Mainardi, Frederic Pene, and Thomas Guillard for their help in drafting the article.

## Funding

The COMBAT study was supported by the Assistance Publique Hopitaux de Paris, French Ministry of Health, Inserm, SPILF, and the Pfizer Pharmaceutical Company, which also funded ORP. The study was endorsed by the following professional organizations: Association Pour l’enseignement de la Thérapeutique, Société de Pathologie Infectieuse de Langue Française, Société Française de Microbiologie, Société Nationale Française de Médecine Interne, Société de Réanimation de Langue Française, Société Française de Gérontologie, Société Française d’Anesthésie-Réanimation.

## Ethics Statement

This study was registered with ClinicalTrials.gov (NCT02916732) and received ethics approval from the Comité de Protection des Personnes Ile de France CPP 4 (IRB 00003835) (2012-16NI) and the French Data Protection Authority (Commission Nationale de l’Informatique et des Libertés) (EGY/FLR/AR128794). In each center, patients were pre-enrolled in the study. Patients or their legal representatives received written information about the study, and only those who provided consent were enrolled. In each center, patients were pre-enrolled in the study. Patients or their legal representatives received written information about the study. Only those who gave consent were definitely enrolled.

## Conflict of Interest

Given his role as Journal of Intensive Medicine, Jean-Pierre Quenot had no involvement in the peer-review of this article and has no access to information regarding its peer-review. Full responsibility for the editorial process for this article was delegated to another journal editor. The authors declare that they have no known competing financial interests or personal relationships that could have appeared to influence the work reported in this paper.

## Data Availability

The datasets from this study are available from the corresponding author upon request.

## CRediT authorship contribution statement

**Valentin de Villiers de la Noue:** Writing – review & editing, Writing – original draft, Validation, Resources, Project administration, Methodology, Investigation, Formal analysis, Data curation, Conceptualization. **Sarah Tubiana:** Validation, Methodology, Formal analysis, Data curation. **Maryam Rontani:** Resources, Methodology, Formal analysis. **Marion Giry:** Writing – review & editing, Data curation. **Benjamine Sarton:** Writing – review & editing, Data curation. **Jean-David Pommier:** Writing – review & editing, Data curation. **Nicolas Terzi:** Writing – review & editing, Data curation. **Mathieu Jozwiak:** Writing – review & editing, Data curation. **Laurent Argaud:** Writing – review & editing, Data curation. **Claire Dupuis:** Writing – review & editing, Data curation. **Nathalie Marin:** Writing – review & editing. **Jean-Pierre Quenot:** Writing – review & editing, Data curation. **Paul Nedelec:** Writing – review & editing, Data curation. **Magali Garcia:** Writing – review & editing, Data curation. **François Barbier:** Writing – review & editing, Data curation. **Khaldoun Kuteifan:** Writing – review & editing, Data curation. **Nacim Benchabane:** Writing – review & editing, Data curation. **Xavier Duval:** Writing – review & editing, Validation, Supervision, Methodology, Formal analysis, Data curation, Conceptualization. **Bruno Mourvillier:** Writing – review & editing, Validation, Supervision, Project administration, Methodology, Investigation, Formal analysis, Data curation, Conceptualization.

## Appendix Supplementary materials

Image, application 1
