# Supplementary material for: Prognostic factors in ICU patients with community-acquired bacterial meningitis: A post hoc analysis of a multicenter prospective cohort study
Source: J Intensive Med. 2026 Jun 13;6(5):524–33. doi: 10.1016/j.jointm.2026.04.007 (PMC13728389; doi:10.1016/j.jointm.2026.04.007)
Supplement: Supplementary file 1 [file mmc1.docx]

| **Supplementary Table 1: Sensitivity analysis: multivariable logistic regression model using ΔSOFA (day 3 minus admission) for 30-day mortality among ICU patients with bacterial meningitis.** | | |
| --- | --- | --- |
| **Variables** | OR [95% CI] | *P* value |
| Δ SOFA (day 3 minus admission) | 1.36 [1.13–1.64] | 0.001 |
| Dexamethasone therapy (yes *vs*. no) | 0.65 [0.23–1.82] | 0.415 |
| Pathogen (*Neisseria meningitidis* *vs*. others) | 0.19 [0.01–3.70] | 0.274 |
| Age group |  | 0.160 |
| - <35 years | Ref. |  |
| - 35–65 years | 1.16 [0.16–8.20] |  |
| - ≥65 years | 2.98 [0.41–21.29] |  |

c-statistic (95% CI): 0.81 [0.72–0.90]; Hosmer–Lemeshow goodness-of-fit: *P*=0.33.

ICU: Intensive care unit; IQR: Interquartile range; SOFA: Sequential Organ Failure Assessment.
